# The effect of different skin-ankle brace application pressures on quiet single-limb balance and electromyographic activation onset of lower limb muscles

**DOI:** 10.1186/1471-2474-8-89

**Published:** 2007-09-12

**Authors:** Emmanuel S Papadopoulos, Christos Nikolopoulos, Athanasios Badekas, George Vagenas, Stamatios A Papadakis, Spyros Athanasopoulos

**Affiliations:** 1 Department of Physical Education and Sports Science, University of Athens, Greece; 2 ORTHO-FOOT CENTER, Department of Podiatry, Nicosia, Cyprus; 3 Department of Orthopaedics, Police Medical Division, Athens, Greece; 4 D' Department of Orthopaedics, "KAT" General Hospital, Kifissia, Greece; 5 28th Octovriou Street, 54, 15236 N. Penteli, Greece

## Abstract

**Background:**

Several studies have been carried out in order to investigate the effect of ankle bracing on ankle joint function and performance. However, no study so far has examined the role of skin-brace interface pressure in neuromuscular control. The aim of this study was to investigate the effect of different skin-ankle brace interface pressures on quiet single limb balance and the electromyographic (EMG) activation sequence of four lower limb muscles.

**Methods:**

Thirty three male physical education students who volunteered to take part in the study were measured under three ankle brace conditions: i) without brace, ii) with brace and 30 kPa application pressure and iii) with brace and 60 kPa application pressure. Single limb balance (anteroposterior and mediolateral parameter) was assessed on the dominant lower limb, with open and closed eyes, on a force platform, simultaneously with the EMG recording of four lower lower limb muscles' (gastrocnemius, peroneus longus, rectus femoris and biceps femoris) activation onset.

**Results:**

The results showed that overall balance (total stability parameter) was not significantly affected in any of the three ankle brace conditions. However, the anteroposterior centre of pressure excursion and centre of pressure excursion velocity were significantly increased with the application of ankle brace, both with 30 and 60 kPa application pressures. Furthermore, it was found that single limb balance was significantly worse with closed eyes compared to open eyes. EMG measurements showed that the sequence of lower limb activation onset was not affected in any of the three ankle brace application conditions. The results of this study showed that the application of an ankle brace with two different skin-brace interface pressures had no effect on overall single limb balance and the sequence of lower limb muscle activation.

**Conclusion:**

These findings suggest that peripheral joint receptors are either not adequately stimulated by the brace application and therefore are not able to alter the balance control strategy of the CNS, or that they play a less important role in the control of single limb balance. Further research is needed in this area with more dynamic and functional measurements, before the safe use of ankle bracing can be widely recommended.

## Background

Ankle bracing comprises one of the most common prophylactic measures used during competition by sports participants, in order to prevent lateral ankle sprains. Several experimental studies have been carried out in order to investigate the effect of ankle bracing on athletic performance and other parameters related to function. However, the effect of ankle bracing on balance and postural control has been investigated by a limited number of studies that have demonstrated both positive and negative findings. Bennell & Goldie [1], showed that application of a Swede-o laced-up brace, and adhesive tape reduced the one-legged stability of uninjured subjects significantly. Similarly, Papadopoulos et al. [2], found that the application of a laced-up ankle brace deteriorated significantly single and double limb balance in young healthy volunteers. It was speculated that this might be due to the restriction of ankle mobility caused by these supports. Therefore, this negative effect may be either due to an inhibiting effect of the brace on peripheral receptors, or to the reduction of ankle range of motion that might interfere with the compensatory balance correcting strategies. On the other hand, Baier & Hopf [3] showed that a rigid and a semirigid ankle brace significantly improved balance in a group of athletes with instability but had no effect on a healthy control group. Furthermore, in another study by Feuerbach and Grabiner [4], it was found that the application of an air-stirrup ankle brace, significantly improved postural control of healthy young subjects, since it reduced both the centre of pressure excursion and centre of pressure excursion velocity during single limb balance.

Friden et al. [5], who also investigated the effect of an air-stirrup ankle brace on the single limb balance of patients with lateral ankle sprains, found no positive or negative effect on centre of pressure excursion and centre of pressure excursion velocity. Likewise, Palmieri et al. [6], who investigated the effect of 4 days ankle-brace use on the mean frequency amplitude of the mediolateral and anteroposterior centre of pressure during one-legged stance, in 28 young healthy college students, found no difference between the brace and control conditions. They concluded that ankle-brace application did not interfere with the proprioceptive control of posture during one-legged stance. However, no information is provided regarding the amount of pressure that the brace was applied by the subjects.

According to all these studies, controversy seems to exist as to the effect of ankle bracing on postural control due to methodological differences of balance assessment, type of brace used and technique application. Furthermore, a limited number of studies have been carried out so far to investigate the effect of ankle bracing on EMG activation time of lower limb muscles. Lower limb activation sequence during standing balance is an important parameter which is related to ankle joint neuromuscular function. Specifically, it was considered important in this study to investigate whether the application of an ankle brace with different pressures affects only the activation sequence of four muscles, and not the overall EMG activity during the 5 sec trial, either due to the stimulation of the skin receptors or to the restriction of joint motion. In a previous study, Roller et al [7] investigated the role of a semirigid ankle brace in the mediolateral and anteroposterior single limb balance, as well as the activation sequence of four lower limb muscles and the abdominals and low back muscles. The results showed no significant difference in AP and ML balance ability and EMG activation time between the conditions with and without brace, which is in aggreement with the findings of the current study. In another study by Rose et al [8], it was found that the application of semirigid orthotic ankle support did not affect the sequence of four knee muscles during dynamic single limb balance in subjects with overpronation of the foot. To the best of our knowledge, these are the only studies that have investigated the role of ankle bracing in lower limb muscles activation sequence, and therefore further research is needed. Furthermore, no studies have investigated or reported what the average brace application pressure is for different brace types. The importance of studying muscle activation onset during a balance task has been demonstrated by several studies [9-11], which described the different strategies used by a person to maintain an upright posture during static [9,10] or dynamic balance tasks [12-17], both in healthy and in idividuals with proprioception deficit. Clearly, there is a need for further research in order to establish the role of brace application pressure on balance and proprioception.

Therefore, the purpose of this study was to investigate the effect of different skin-brace interface application pressures on quiet single-limb static balance and whether the balance maintenance strategies during a single limb stance, can be altered with the application of a specific widely used type of ankle brace the sequence of lower limb muscle activation during balance.

## Methods

Thirty-three male physical education students, volunteered to take part in the study. The experimental investigation with human subjects reported in the manuscript was performed with informed consent and followed all the guidelines for experimental investigation with human subjects required by the institutional review board and the ethics committee with which the principal investigators are affiliated. Subjects had no history of severe ankle sprains and joint instability and did not ever make use of an ankle brace or any other type of ankle support. Eligible subjects that were entered to the study underwent anthropometric measurements (Table 1), and were followed by the assessment of the single limb balance with open and closed eyes in conjunction with the EMG activation time measurements, under three conditions: i) without brace; ii) with brace and 30 kPa application pressure; and iii) with brace and 60 kPa application pressure. A moderate and a high application pressure were chosen because practically, athletes apply ankle braces subjectively, according to the level of support and comfort they prefer. Comfort means better performance and support better injury prevention and the criterion for this choice lies with the individual so far, since no study exists to support any of these parameters. Brace application pressure of 30 kPa resembles a moderately tightened brace and may be chosen by some sports participants in order to be more comfortable and to not hinder their performance. On the other hand, 60 kPa brace application resembles a highly tight application with none of the subjects however reporting pain, discomfort, discolouration or microcirculation disturbances, in this study. This tight application may be chosen by sports participants whose main concern is to prevent ankle injury or re-injury. Furthermore, 60 kPa application pressure is safe because it is significantly lower than the skin pressure threshold of 100 kPa which causes skin breakage [20]. Since ankle brace application pressures have not been measured by previous studies, this is a first attempt to apply pressures as close as possible to the commonly used application techniques by sports participants, for the above reasons.

**Table 1:** Demographic variables (N = 33)

**Age**	21.5 ± 1.5
**Weight (kgrs)**	77.9 ± 8.9
**Height (cm)**	177.5 ± 7.4
**BMI**	24.8 ± 2.9
**Fat (%)**	19.9 ± 4.1
**Lean Body Mass**	62.2 ± 6.1
**Activity level**	8.5 ± 1.5

The laced-up McDavid ankle brace (McDavid Ankle Guard Inc., Chicago, IL) was used for the measurements in the study.

### Single limb balance assessment

Single limb balance was assessed using the MatSCAN force platform (Tekscan Inc., Boston, MA) which displayed the average centre of pressure (COP) excursion and centre of pressure excursion velocity in the anteroposterior and mediolateral directions. All measurements described in the study were performed without shoes. All measurements, in all subjects, were performed firstly without brace, secondly with brace and 30 kpa pressure and thirdly with brace and 60 kPa pressure. For this reason an order effect was ruled out, since it was the same for all subjects. The Tekscan mat calibration was done using a Uniform Bladder System, described by Nicolopoulos [18].

A combined stability parameter (*σ*_r_), described by Riley et al. [19], was also used for the overall balance assessment, which is based on the root mean square variance of both the centre of pressure excursion and centre of pressure excursion velocity in the anteropostreior and mediolateral direction according to the formula:

*σ*_*r *_= *√σ*_*AP*_^2^_*r *_+ *σ*_Latr_^2^_r_

where: *σ*_*r *_is the combined stability parameter; *σ*_*AP*_^2^_*r *_and *σ*_Latr_^2^_r _are the directional anteroposterior and mediolateral stability parameters correspondingly.

**Figure 1:**
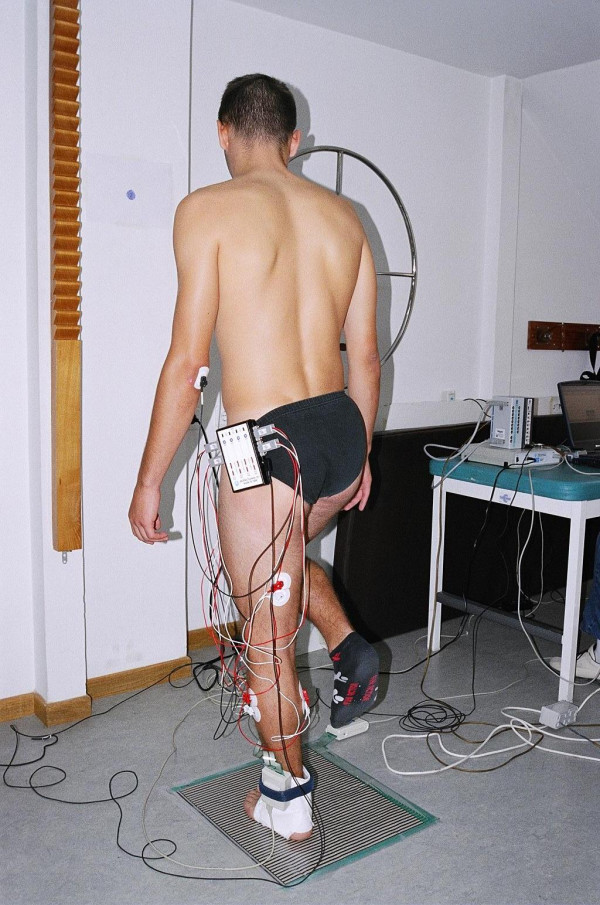
Single limb balance and muscle activation measurement.

The stability parameter was calculated in order to have a picture of the brace application on the total balance. Subjects were asked to stand on their dominant foot (which was determined by asking them to pretend to kick a ball, with the kicking leg being the dominant), for 5 seconds as quietly as possible, staring at a 3 cm spot fixed one meter on the wall in front of them (Figure 1). Balance was measured with open and closed eyes and a mean of two trials for each condition was calculated. The same procedure was repeated for all three ankle brace conditions.

Interface pressure was measured with the 9811 F-Socket (Teckscan Inc., Boston, MA) sensor which was applied in the anterior aspect of the ankle underlying the brace laces. The F-Socket was calibrated using a sphygmomanometer around the ankle joint up to the point where the desired pressures (30 & 60 kPa) were reached.

**Figure 2:**
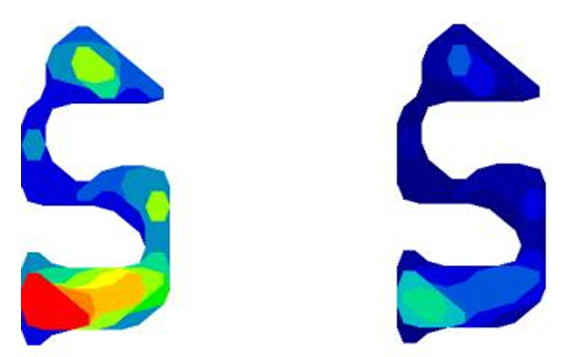
Skin-ankle brace interface pressure pattern for 60 kPa (left) and 30 kPa (right) pressure application conditions.

Pressure was applied by tightening the laces until the predetermined amount of pressure (30 & 60 kPa) was reached (Figure 2). The two pressures were chosen because according to Convery & Bui [20], any pressure of 100 kPa and above can cause skin damage, therefore the higher pressure of 60 kPa was much below that level and the 30 kPa was chosen as a moderate pressure. Furthermore, according to Meinders et al [21], pressures of 40 kPa and over can temporarily stop microcirculation but cause immediate hyperaemia immediately after pressure is removed. Since the brace was removed after each experiment there was no incident of skin irritation or damage.

### EMG measurements

The effect of the McDavid ankle brace on the sequence of lower limb EMG muscle activation was measured simultaneously with the balance measurements. Surface Electromyography (EMG) was used to determine the activation time of the gastrocnemius, peroneous longus, rectus femoris and biceps femoris muscles, using a pair of bipolar surface silver chloride electrodes. These muscles were selected because according to the literature they have been used by previous studies for the EMG measurement during single limb balance and are of significant importance in the control strategy of standing balance [10,17].

The Biopac MP100 System (BiopacSystems, Inc, Goleta, CA) was used to record and analyse the EMG signal. After the skin was shaved and degreased with 70% alcoholic solution, electrodes were attached to the skin parallel to the muscle fibres, on the most prominent point of the muscles [22-24], during isometric contraction and palpation, according to the specifications by Perotto [24], and an interelectrode distance of 3 cm. Correct placement of the electrodes was tested for crosstalk by asking the subjects to perform active contractions of all four muscles that were measured. The ground electrodes ware placed on bony prominences of the knee and ankle. The raw EMG signal was sampled by the computer with a frequency of 1000 Hz. Processing of the raw EMG signal was performed by converting it to RMS, band pass filtrered between 20 and 500 Hz, in order to subtract electromagnetic noise, and movement artefacts were filtered with a high pass cut off frequency of 20 Hz [25]. The first visible signal that was 2 SDs above baseline activity was considered as the onset of muscle activation [12,15,16].

### Statistical analysis

Statistical analysis was performed with the SPSS version 11.5. In order to analyze differences for balance and EMG measurements between the three ankle brace conditions, as well as for open and closed eyes and the eyes by brace interactions, the two way ANOVA was used. The Bonferonni test was applied for post-hoc comparisons in order to calculate the range of differences and the mean differences, together with the 95% confidence interval. The paired t-test and the mixed effects ANOVA model on the other hand were used to calculate differences for the stability parameter. Since balance was measured twice on the same individual, once with open and another time with closed eyes, a paired t-test was used to calculate the difference in stability parameter between open and closed eyes.

Similarly, as the balance for the 3 ankle brace conditions was measured three times on the same individual, a mixed effects ANOVA model was used to calculate the differences in the stability parameter between the three brace conditions taking also into account the correlation between adjacent observations. For the assessing the statistical significance of a hypothesis a common significance level of 5% was assumed.

## Results

### Single limb balance

Mean values for the centre of pressure excursion (mm) and centre of pressure excursion velocity (mm/sec), in the mediolateral and anteroposterior direction are displayed in Tables 2 and 3. No significant differences were found in the mediolateral centre of pressure excursion and centre of pressure excursion velocity between the three ankle brace conditions (F = 0.29, df = 2, p = 0.749) (Table 2).

**Table 2:** Mean values for mediolateral sway and sway velocity for three ankle brace conditions with open and closed eyes (N = 33)

	**Medioliateral sway (mm)**		**Medioliateral sway velocity (mm/sec)**	
	
	Open eyes	Closed eyes	Open eyes	Closed eyes
**Ankle brace condition**	x¯ MathType@MTEF@5@5@+=feaafiart1ev1aaatCvAUfKttLearuWrP9MDH5MBPbIqV92AaeXatLxBI9gBaebbnrfifHhDYfgasaacH8akY=wiFfYdH8Gipec8Eeeu0xXdbba9frFj0=OqFfea0dXdd9vqai=hGuQ8kuc9pgc9s8qqaq=dirpe0xb9q8qiLsFr0=vr0=vr0dc8meaabaqaciaacaGaaeqabaqabeGadaaakeaacuqG4baEgaqeaaaa@2E3B@ ± SD	x¯ MathType@MTEF@5@5@+=feaafiart1ev1aaatCvAUfKttLearuWrP9MDH5MBPbIqV92AaeXatLxBI9gBaebbnrfifHhDYfgasaacH8akY=wiFfYdH8Gipec8Eeeu0xXdbba9frFj0=OqFfea0dXdd9vqai=hGuQ8kuc9pgc9s8qqaq=dirpe0xb9q8qiLsFr0=vr0=vr0dc8meaabaqaciaacaGaaeqabaqabeGadaaakeaacuqG4baEgaqeaaaa@2E3B@ ± SD	x¯ MathType@MTEF@5@5@+=feaafiart1ev1aaatCvAUfKttLearuWrP9MDH5MBPbIqV92AaeXatLxBI9gBaebbnrfifHhDYfgasaacH8akY=wiFfYdH8Gipec8Eeeu0xXdbba9frFj0=OqFfea0dXdd9vqai=hGuQ8kuc9pgc9s8qqaq=dirpe0xb9q8qiLsFr0=vr0=vr0dc8meaabaqaciaacaGaaeqabaqabeGadaaakeaacuqG4baEgaqeaaaa@2E3B@ ± SD	x¯ MathType@MTEF@5@5@+=feaafiart1ev1aaatCvAUfKttLearuWrP9MDH5MBPbIqV92AaeXatLxBI9gBaebbnrfifHhDYfgasaacH8akY=wiFfYdH8Gipec8Eeeu0xXdbba9frFj0=OqFfea0dXdd9vqai=hGuQ8kuc9pgc9s8qqaq=dirpe0xb9q8qiLsFr0=vr0=vr0dc8meaabaqaciaacaGaaeqabaqabeGadaaakeaacuqG4baEgaqeaaaa@2E3B@ ± SD
Without brace	36.04 ± 23.2	52.8 ± 28.0*	290.2 ± 184.2	423.6 ± 224.2*
With brace (30 kPa)	34.2 ± 22.7	48.5 ± 21.2*	275.06 ± 183.7	401.0 ± 185.5*
With brace (60 kPa)	35.2 ± 23.8	50.9 ± 19.2*	284.03 ± 188.8	407.4 ± 153.6*

* F = 22.3, df = 1, p < 0.001 for the difference between open and closed eyes

**Table 3:** Mean values for the anteroposterior sway and sway velocity for three ankle brace conditions with open and closed eyes (N = 33)

	**Anteropostreior sway (mm)**		**Anteroposterior sway velocity (mm/sec)**	
	
	Open eyes	Closed eyes	Open eyes	Closed eyes
**Ankle brace condition**	x¯ MathType@MTEF@5@5@+=feaafiart1ev1aaatCvAUfKttLearuWrP9MDH5MBPbIqV92AaeXatLxBI9gBaebbnrfifHhDYfgasaacH8akY=wiFfYdH8Gipec8Eeeu0xXdbba9frFj0=OqFfea0dXdd9vqai=hGuQ8kuc9pgc9s8qqaq=dirpe0xb9q8qiLsFr0=vr0=vr0dc8meaabaqaciaacaGaaeqabaqabeGadaaakeaacuqG4baEgaqeaaaa@2E3B@ ± SD	x¯ MathType@MTEF@5@5@+=feaafiart1ev1aaatCvAUfKttLearuWrP9MDH5MBPbIqV92AaeXatLxBI9gBaebbnrfifHhDYfgasaacH8akY=wiFfYdH8Gipec8Eeeu0xXdbba9frFj0=OqFfea0dXdd9vqai=hGuQ8kuc9pgc9s8qqaq=dirpe0xb9q8qiLsFr0=vr0=vr0dc8meaabaqaciaacaGaaeqabaqabeGadaaakeaacuqG4baEgaqeaaaa@2E3B@ ± SD	x¯ MathType@MTEF@5@5@+=feaafiart1ev1aaatCvAUfKttLearuWrP9MDH5MBPbIqV92AaeXatLxBI9gBaebbnrfifHhDYfgasaacH8akY=wiFfYdH8Gipec8Eeeu0xXdbba9frFj0=OqFfea0dXdd9vqai=hGuQ8kuc9pgc9s8qqaq=dirpe0xb9q8qiLsFr0=vr0=vr0dc8meaabaqaciaacaGaaeqabaqabeGadaaakeaacuqG4baEgaqeaaaa@2E3B@ ± SD	x¯ MathType@MTEF@5@5@+=feaafiart1ev1aaatCvAUfKttLearuWrP9MDH5MBPbIqV92AaeXatLxBI9gBaebbnrfifHhDYfgasaacH8akY=wiFfYdH8Gipec8Eeeu0xXdbba9frFj0=OqFfea0dXdd9vqai=hGuQ8kuc9pgc9s8qqaq=dirpe0xb9q8qiLsFr0=vr0=vr0dc8meaabaqaciaacaGaaeqabaqabeGadaaakeaacuqG4baEgaqeaaaa@2E3B@ ± SD
Without brace	14.8 ± 3.09	25.64 ± 7.99**	118.06 ± 24.08	205.12 ± 63.9**
With brace (30 kPa)	15.39 ± 3.99	31.23 ± 9.88**	123.15 ± 31.9	249.8 ± 79.02**
With brace (60 kPa)	16.41 ± 4.44	30.24 ± 10.1**	131.45 ± 35.1	241.88 ± 80.5**

** F = 22.8, df = 1, p < 0.001 for the difference between open and closed eyes in Velocity

In the anteroposterior direction however, significant differences were found between the condition with 30 kPa brace application and the condition without brace (mean difference = 3.08, 95% CI: 0.06–6.1; p = 0.043) and between the condition with 60 kPa brace application and the condition without brace (mean difference = 3.09, 95% CI: 0.08–6.11; p = 0.042). Specifically, ankle brace application resulted in a deterioration of the anteroposterior COP excursion and excursion velocity, both with open and closed eyes (Table 3). As far as the effect of vision is concerned, significant differences were found between open and closed eyes, both in the mediolateral (F = 22.3, df = 1, 192, p < 0.001) and the anteroposterior (F = 175.4, df = 1, 192, p < 0.001) direction, in all three ankle brace application pressure conditions, with single limb balance being significantly worse with closed eyes. (Tables 2 and 3).

As far as the stability parameters are concerned, no significant differences were detected in single limb balance between the three brace application conditions, in the anteroposterior (F = 2.12, df = 2, p = 0.13), mediolateral (F = 0.05, df = 2, p = 0.95) and the total (F = 0.26, df = 1, p = 0.77) stability parameters. Lastly, significant differences were found in the total stability parameter between open and closed eyes (F = 2.24, df = 32, p = 0.032). Table 4 displays the mean values and standard deviations of the stability parameters for the three ankle brace and eye conditions.

**Table 4:** Mean values for the anteroposterior (*σ*_Apr_), mediolateral (*σ*_Latr_) and total (*σ*_r_) stability parameters for open and closed eyes and the three ankle brace conditions (N = 33)

**Condition**	*σ*_Apr_	*σ*_Latr_	*σ*_r_
Open eyes	21.83 ± 11.4	131.65 ± 103.3	136.41 ± 99.9
Closed eyes	69.9 ± 39.5	152.17 ± 85.3	174.45 ± 79.87
Without brace	65.84 ± 45.5	116.09 ± 119.3	145.2 ± 113.7
With brace 30 kPa	92.68 ± 54.8	118.71 ± 90.12	162.5 ± 85.3
With brace 60 kPa	79.05 ± 54.8	124.08 ± 94.6	157.3 ± 93.7

### EMG Measurements

Analysis of variance showed that there were no significant differences in the activation time of the peroneus longus (F = 0.008, df = 2, p = 0.99), gastrocnemius (F = 0.28, df = 2, p = 0.75), rectus femoris (F = 1.13, df = 2, p = 0.32) and biceps femoris (F = 2.11, df = 2, p = 0.124), between the three ankle brace application conditions. Furthermore, no significant differences in EMG activation time were found in single limb balance between open and closed eyes for the peroneus longus, gastrocnemius and rectus femoris muscles. However, significantly faster activation of the biceps femoris muscle was detected with open eyes as compared to closed eyes, in all three brace conditions. Mean EMG activation time values are displayed in Table 5. No change in the EMG activation time sequence was observed for the four lower limb muascles that were tested in the three brace application conditions (Table 6).

**Table 5:** Mean EMG activation times (msec), of lower limb muscles, for the three ankle brace conditions with open (OE) and closed eyes (CE) (N = 33)

	**Peroneous longous**		**Gastrocnemius**		**Rectus Femoris**		**Biceps Femoris**	
Ankle brace condition	OE	CE	OE	CE	OE	CE	OE	CE

Without brace	31.7 ± 6.3	30.7 ± 6.4	33.06 ± 7.07	32.3 ± 6.4	117.06 ± 31.2	117.87 ± 35.9	118.5 ± 27.1	123.8 ± 34.7
With brace (30 kPa)	31.2 ± 5.2	31.1 ± 6.8	32.2 ± 5.4	31.5 ± 6.7	111.8 ± 22.1	125.1 ± 28.4	118.8 ± 26.4	143.1 ± 42.8
With brace (60 kPa)	31.6 ± 5.5	30.5 ± 6.3	32.5 ± 7.3	32.2 ± 6.1	127.8 ± 35.1	121.69 ± 26.2	119.2 ± 27.5	123.5 ± 25.4

**Table 6:** EMG activation sequence of lower limb muscles during single limb balance in the three brace application conditions

**Brace condition**	EYES	**Peroneus longus**	**Gastrocnemius**	**Rectus femoris**	**Biceps femoris**
Without brace	Open	1	2	3	4
	Closed	1	2	3	4
With brace (30 kPa)	Open	1	2	3	4
	Closed	1	2	3	4
With brace (60 kPa)	Open	1	2	4	3
	Closed	1	2	3	4

## Discussion

To the best of our knowledge, this study is the first to investigate the effects of ankle brace application pressures on postural control and electromyographic activation sequence of lower limb muscles. In a recent study, Papadopoulos et al. [26], examined the effects of different ankle brace application pressures on the peroneus longus reaction time, during a sudden inversion stress test and found that ankle brace application with medium and high pressure, resulted in a significant delay of the peroneal reaction time. In the current study, we investigated the effect of no brace application and two different ankle brace application pressures, on single limb balance control and the electromyographic activation sequence of four lower limb muscles. The results showed that overall with the specific type of brace that was used in this study, postural control, as assessed by the total stability parameter '*σ*_r_', was not positively or adversely affected by the two different brace application pressures. This finding is in agreement with previous studies, which showed that ankle bracing had no effect on postural control, without however, referring to the pressure of brace application [5,6]. It was also shown that different ankle brace application pressures had no effect on the mediolateral plane but on the other hand, it significantly deteriorated balance in the anteroposterior plane. These findings are partly in agreement with Bennell & Goldie [1], and Papadopoulos et al. [2], who also found that ankle bracing adversely affected balance in young healthy volunteers. However, the fact that mediolateral balance was not affected in any of the three brace application conditions may be more valid in this research since in this study sample, the COP trajectory during single limb balance, mainly traveled in the mediolateral direction. The deterioration of balance in the anteroposterior direction may also be attributed to the fact that muscular control of single limb balance which is more efficient in the AP direction [27], was adversely affected by the application of the ankle brace. Furthermore, since both the mediolateral (*σ*_Apr_) and the anteroposterior (*σ*_Latr_) stability parameters, as well as the total stability parameter (*σ*_r_), were not significantly affected, it may be concluded that overall, the application of the two different ankle brace application pressures, had no positive or negative effect on quiet single limb balance. This is also supported by Riley et al. [7], who stated that the stability parameter they calculated, which combines both the centre of pressure excursion and centre of pressure excursion velocity, as well as the mediolateral and anteroposterior planes, is more valid and representative for the assessment of standing balance than separately assessing each of these parameters alone. This information may be useful in future studies as well as in the clinical setting, since it seems that the application of the laced-up ankle brace with a moderate and a high pressure, had no significant stimulation effect on the peripheral, mainly skin receptors, and therefore afferent signals were not strong enough to provoke a specific central response and affect single limb balance control. Another finding of this study was that single limb balance was significantly worse with closed eyes as compared to open eyes. This is in agreement with previous studies [2,28-31] and further establishes the importance of vision which is one of the three major sources of balance control together with the vestibular system and the peripheral joint receptors [32].

The results of the electromyographic measurements showed that the sequence of lower limb muscle activation onset was not altered by the application of two different ankle brace application pressures. This finding cannot be compared with previous studies since this is the only study that has investigated the effect of ankle bracing on the EMG activity of lower limb muscles and, as mentioned above, the only that has examined the effect of different brace application pressures. However, useful information that arises is that different ankle brace application pressures do not change the ankle strategy of balance control, which is the one that dominates during balance of young healthy subjects [15,10,14,9,33]. Therefore, the fact that the more distal peroneus longus and gastrocnemius muscles were activated faster than the more proximal thigh muscles (rectus femoris and biceps femoris), both in the condition without brace and the conditions with moderate and high brace application pressures, further supports the hypothesis that the central nervous system (CNS) does not alter its single limb balance control strategy. Several explanations may be given for this observation: first, the skin receptors are either not adequately stimulated by the high application pressure of the brace so as, in turn, to provoke a CNS response, or their role in controlling single limb balance is less important. Second, the fact that the study sample consisted of non-injured subjects, may in part explain the lack of pressure application effect on balance. It could be argued that joint receptors in healthy subjects may be adequate in preventing single balance distortion with different ankle brace application pressures. However, this is not supported by previous studies since ligament receptors are stimulated in the end ranges of joint motion and therefore ligament injury affects only the mechanical and not the functional dynamic stability of the joint which is mostly controlled by the muscle spindles [32]. Further research, which will investigate the effect of ankle brace application in injured subjects too, is necessary.

## Conclusion

The findings of this study showed that regardless of application pressure, ankle bracing had no adverse effect in single limb balance or EMG activation sequence. This finding is limited to the specific type of ankle brace that was used in the study and cannot be generalized to other types of ankle support. However, in another study by Papadopoulos et al. [26], it was found that both moderate and high application pressures delayed the activation of the peroneus longus during sudden inversion. That fact that this is the only active protective mechanism against lateral ankle sprains questions the safety of wide ankle brace use and necessitates the need for further research. This will include performance measurements as well as the comparison of different types of ankle supports including rigid ankle braces in more real functional sports related activities, in order to see if their increased restricting effect (shown in previous studies), interfere with function and performance. The findings of this study which refer to the prophylactic use of the laced up ankle brace, may contribute to the determination of the optimum application technique in order to combine prevention and performance.

## Competing interests

The authors declare that they have no competing interests.

## Authors' contributions

ESP, CN, and AB participated in the design of the study, data acquisition and analysis and writing of this manuscript. GV, participated in the analysis and writing of this paper. SAP and SA participated in the analysis and also in revising critically the manuscript. All authors read and approved the final manuscript.

## Pre-publication history

The pre-publication history for this paper can be accessed here:



## References

[B1] Bennell KL, Goldie PA (1994). The differential effects of external ankle support on postural control. J Orthop Sport Phys Ther.

[B2] Papadopoulos E, Karzis K, Tsakoniti K, Karteroliotis K, Athanasopoulos S (2002). The immediate effect of ankle bracing, head extension and vision on single limb balance. Proceedings of the 7th Congress of the European College of Sport Science: 24–28 July 2002; Athens.

[B3] Baier M, Hopf T (1998). Ankle orthoses effect on single-limb standing balance in athletes with functional ankle instability. Arch Phys Med Rehab.

[B4] Feuerbach JW, Grabiner MD (1993). Effect of the aircast on unilateral postural control: amplitude and frequency variables. J Orthop Sport Phys Ther.

[B5] Friden T, Zatterstrom R, Moritz U (1989). A stabilometric technique for evaluation of lower limb instabilities. Am J Sport Med.

[B6] Palmieri RM, Ingersoll CD, Cordova ST, Kinzey SJ (2002). The spectral qualities of postural control are unaffected by 4 days of ankle-brace application. J Athl Training.

[B7] Roller SJ, Livengood AL, Mattacola CG, Uhl TL, Malone TR (2003). Effect of prophylactic ankle bracing on postural control and EMG of lower extremity and trunk muscles. Journal of Athletic Training.

[B8] Rose HM, Shultz SJ, Arnold BL, Gansneder BM, Perrin DH (2002). Acute orthotic intervention does not affect muscular response times and activation patterns at the knee. Journal of Athletic Training.

[B9] Riemann BL, Myers JB, Lephart SM (2003). Comparison of the ankle, knee, hip and trunk corrective action shown during single-leg stance on firm, foam and multiaxial surfaces. Arch Phys Med Rehab.

[B10] Gatev P, Thomas S, Kepple T, Hallet M (1999). Feedforward ankle strategy of balance during quiet stance in adults. J Physiol.

[B11] Carpenter MG, Frank JS, Silcher CP, Peysar GW (2001). The influence of postural threat on the control of upright stance. Experimental Brain Research.

[B12] Henry SM, Joyce F, Horak F (1998). EMG responses to maintain stance during multidirectional surface translations. J Neurophysiol.

[B13] Gilles M, Wing AM, Kirker SGB (1999). Lateral balance organization in human stance in response to a random or predictable perturbation. Experimental Brain Research.

[B14] King DL, Zatsiorsky VM (2002). Periods of ankle displacement during one-legged standing. Gait Posture.

[B15] Bloem BR, Allum JHJ, Carpenter MG, Honegger F (2000). Is lower leg proprioception essential for triggering automatic postural responses?. Exp Brain Res.

[B16] Bloem BR, Allum JHJ, Carpenter MG, Verschuuren JJGM, Honegger F (2002). Triggering of balance corrections and compensatory strategies in a patient with total leg proprioceptive loss. Exp Brain Res.

[B17] Allum JHJ, Bloem BR, Carpenter MG, Honegger F (2001). Differential diagnosis of proprioceptive and vestibular deficits using dynamic support-surface posturography. Gait and Posture.

[B18] Nicolopoulos CS (2002). Diagnosis of the Normal and Pathological Foot using the Plantar Preessure Measurements. MSc Thesis.

[B19] Riley PO, Benda BJ, Gill-Body KM, Krebs DE (1995). Phase plane analysis of stability in quiet standing. J Rehabil Res Dev.

[B20] Convery P, Buis AWP (1999). Socket/stump interface dynamic pressure distributions recorded during the prosthetic stance phase of gait of trans-tibial amputee wearing a hydrocast socket. Prosthetics and Orthotics International.

[B21] Meynders MJ, de Lange A, Netten PM, Wollesheim H, Lutterman JA (1996). Micricirculation in the footsole as a function of mechanical pressure. Clinical Biomechanics.

[B22] Konradsen L, Voigt M, Hojsgaard C (1997). Ankle inversion injuries: The role of the Dynamic Defense Mechanism. Am J Sport Med.

[B23] Lynch SA, Eklund U, Gottlieb D, Rensrom AFH, Beynnon B (1996). Electromyographic latency changes in the ankle musculature during inversion moments. Am J Sport Med.

[B24] Perotto A (1996). Anatomical guide for the electromyographer. The trunk and lower limbs.

[B25] De Luca CJ Surface electromyography detection and recording. http://nmrc.bu.edu/nmrc/detect/emg.htm#general.

[B26] Papadopoulos ES, Nicolopoulos C, Baldoukas A, Anderson EG, Athanasopoulos S (2005). The effect of different ankle brace-skin application pressures on the electromyographic peroneus longus reaction time. The Foot.

[B27] Loram ID, Maganaris CN, Lakie M (2005). Human postural sway results from frequent, ballistic bias impulses by soleus and gastrocnemius. Journal of Physiology.

[B28] Bernier JN, Perrin DH (1998). Effect of coordination training on proprioception of the functionally unstable ankle. J Orthop Sport Phys Ther.

[B29] Goldie PA, Bach TM, Evans OM (1989). Force platform measures for evaluating postural control: reliability and validity. Arch Phys Med Rehab.

[B30] Goldie PA, Evans OM, Bach TM (1992). Steadiness in one-legged stance: development of a reliable force-platform testing procedure. Arch Phys Med Rehab.

[B31] Perrin PP, Bene MC, Durupt D (1997). Ankle trauma significantly impairs posture-a study in basketball players and controls. Int J Sports Med.

[B32] Lephart SM, Pincivero DM, Rozzi SL (1998). Proprioception of the ankle and knee. Sports Med.

[B33] Yaggie JA, McGregor SJ (2002). Effects of isokinetic ankle fatigue on the maintenance of balance and postural limits. Arch Phys Med Rehab.

